# Three-dimensional microangiography of the mouse brain using super-resolution ultrasound and optoacoustic imaging with a spherical array transducer^☆^

**DOI:** 10.1016/j.pacs.2026.100823

**Published:** 2026-03-28

**Authors:** Daniil Nozdriukhin, Yi Chen, Cristian Ciobanu, Elshad Feyzili, Daniel Razansky, Xosé Luís Deán-Ben

**Affiliations:** a Institute for Biomedical Engineering and Institute of Pharmacology and Toxicology, Faculty of Medicine, University of Zürich, Winterthurerstrasse 190, Zürich, 8057 Switzerland; b Institute for Biomedical Engineering, Department of Information Technology and Electrical Engineering, ETH Zürich, Wolfgang-Pauli-Strasse 27, Zürich, 8093 Switzerland

**Keywords:** Localization Optoacoustic Tomography, Ultrasound Localization Microscopy, Brain imaging

## Abstract

High-resolution visualization of the mouse brain microvasculature is essential for advancing neurovascular research and understanding neurological disorders. Recent advances in pulse-echo ultrasound (US) and optoacoustic (OA) imaging enable angiographic imaging beyond the acoustic diffraction limit through localization of microbubbles in ultrasound localization microscopy (ULM) and light-absorbing microparticles in localization optoacoustic tomography (LOT). Despite their distinct contrast mechanisms, a direct comparison has been lacking. Here, we evaluate three-dimensional motion-contrast US and OA imaging, including their super-resolved variants ULM and LOT, using the same ultrasound array, localization and tracking algorithms, and frame rates. Studies in mice of different ages reveal complementary strengths: OA/LOT offers higher-SNR for cortical imaging, especially in older animals with thicker skulls, while the lower attenuation of ultrasound enables US/ULM to achieve substantially greater penetration depth and whole-brain coverage. This comparison provides practical guidance for choosing optimal localization-based strategies for cerebrovascular studies.

## 1 Introduction

The ability to visualize mouse brain microvasculature is fundamental for deciphering how the cerebrovascular network supports neural function and metabolism at the microscopic level [1], [2]. Detailed vascular mapping is essential not only for characterizing the structural organization of the brain but also for investigating pathological alterations, e.g. associated with stroke, neurodegeneration, or aging [3], [4], [5]. Moreover, it provides a critical framework for assessing blood–brain barrier (BBB) integrity and neurovascular coupling [3], [4]. Importantly, the availability of numerous mouse models that recapitulate key phenotypes of human diseases further underscores the value of mice as indispensable translational systems for developing and testing therapies targeting cerebrovascular and neurological disorders [5], [6].

Pulse-echo ultrasound (US) and optoacoustic (OA, photoacoustic) imaging have demonstrated complementary capabilities for visualizing the murine brain, particularly at a functional level [7], [8], [9]. Both modalities can map hemodynamic changes associated with neuronal activity via neurovascular coupling, with US providing blood flow information and OA revealing the biodistributions of oxygenated and deoxygenated hemoglobin [10]. Recent developments have further advanced both methods to enable super-resolution angiographic imaging, overcoming the acoustic diffraction limit through localization and tracking of microbubbles or light-absorbing microparticles. Localization-based imaging capitalizes on the sparse, random distribution of intravenously injected particles to reconstruct high-resolution maps of the microvasculature and retrieve blood velocity readings via particle tracking over multiple frames. Since its first *in vivo* demonstration roughly a decade ago, ultrasound localization microscopy (ULM) has been applied in numerous preclinical and clinical studies, delivering structural and hemodynamic insights down to the capillary level (∼10–20 µm resolution) [11], [12], [13], [14]. More recently, localization optoacoustic tomography (LOT), using strongly absorbing microparticles, has shown similar super-resolution capabilities, overcoming both light diffusion and acoustic diffraction barriers [15], [16]. The optical-absorption contrast of OA enables LOT to simultaneously generate high-resolution angiographic maps, blood velocity measurements, and oxygen saturation maps, offering a unique, comprehensive tool for microvascular characterization [15], [17].

The motion-contrast US (MC-US) and motion-contrast OA (MC-OA) imaging and their super-resolved extensions (ULM and LOT) share a common processing framework involving the injection of microparticulate agents, clutter filtering, image compounding or sub-pixel localization to generate angiograms. However, their underlying physical contrast mechanisms result in distinct advantages and limitations. US exhibits weaker attenuation in biological tissues compared to light [18], thus attaining superior penetration depth, e.g. whole-brain imaging in rodents [19]. In contrast, OA benefits from unidirectional wave propagation and the availability of microparticles that can yield exceptional signal-to-noise ratio (SNR) in superficial cortical regions [15], [17]. High SNR is a critical determinant of final image resolution in localization microscopy [20], [21]. Furthermore, the bidirectional propagation of waves in pulse-echo US can induce strong aberrations in acoustically heterogeneous regions like the skull, a challenge less pronounced in OA. These complementary physical principles suggest that the choice between MC-US/ULM and MC-OA/LOT is not trivial and may be highly dependent on the specific experimental requirements, such as the target brain region and the age of the animal, which affects skull properties.

Both MC-US/ULM and MC-OA/LOT embodiments capable of rendering three-dimensional images have been proposed [22], [23], which is essential for blood velocity mapping and structural analysis. In this work, we present a rigorous, side-by-side evaluation of 3D MC-US/ULM and MC-OA/LOT imaging of the mouse brain. The study was conducted on animals of different ages to account for varying skull properties. Identical hardware and processing pipelines were considered to isolate the impact of the fundamental physical principles of each modality. The aim is to establish a practical framework for choosing the optimal super-resolution strategy based on specific experimental needs, such as cortical resolution versus whole-brain coverage.

## 2 Results

### 2.1 Ultrasound and optoacoustic microangiography with a spherical array transducer

**Fig. 1:**
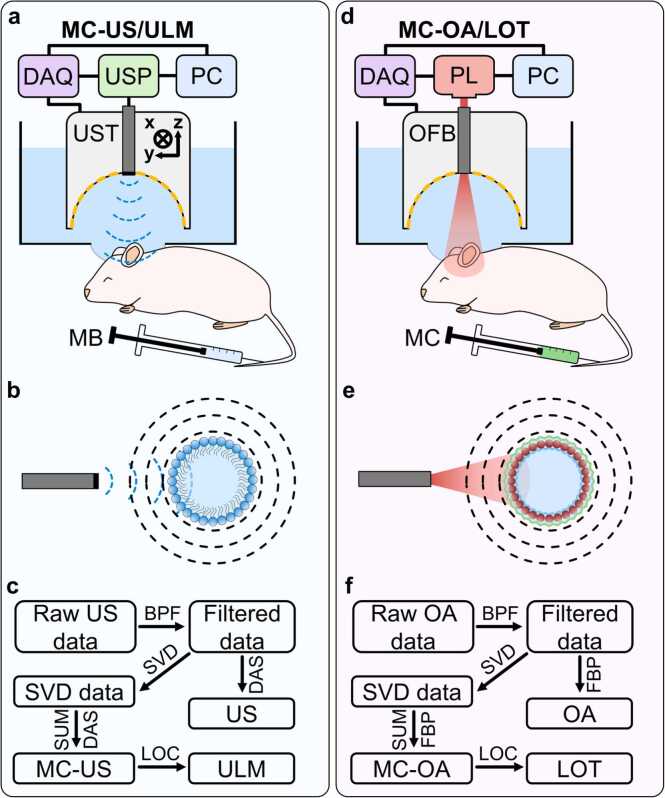
a) General scheme of the 3D motion-contrast ultrasound (MC-US) and ultrasound localization microscopy (ULM) imaging setup. DAQ – multichannel data acquisition system; USP – ultrasound pulser, connected to a single-element ultrasound transducer (UST), inserted into the central aperture of the spherical ultrasound array transducer; PC – personal computer; MB – microbubble suspension. b) Principle of ultrasound (US) contrast-enhanced imaging, based on inducing a vibration of a phospholipid-shell microbubble with US pulses. c) Processing pipeline of the acquired US data. BPF – Butterworth bandpass filter; SVD – singular value decomposition filtering; DAS – delay-and-sum beamforming; SUM – summation of the normalized volumetric images; LOC – localization. d) General scheme of the 3D motion-contrast optoacoustic (MC-OA) and localization optoacoustic tomography (LOT) imaging setup. DAQ – multichannel data acquisition system; PL – nanosecond pulsed laser, guided via an optic fiber bundle (OFB) inserted into the central aperture of the spherical ultrasound array transducer; PC – personal computer; MC – microcapsule suspension. e) Principle of optoacoustic (OA) contrast-enhanced imaging, based on light absorption and conversion of the laser pulse energy into an emitted ultrasound signal at the microcapsule shell. f) Processing pipeline of the acquired OA data. BPF – Butterworth bandpass filter; SVD – singular value decomposition filtering; FBP – filtered back-projection reconstruction; SUM – summation of the normalized volumetric images; LOC – localization.

A unified imaging platform was developed to enable direct comparison between 3D MC-US/ULM and MC-OA/LOT (Fig. 1a,d). In both configurations, signal acquisition was performed using a custom-built 512-element spherical US array. For MC-US/ULM, a single-element 7 MHz transducer (US emitter) connected to an US pulser was inserted coaxially through the central aperture of the array to deliver acoustic excitation pulses (Fig. 1a). The resulting acoustic responses from circulating microbubbles were detected simultaneously across all array elements and digitized by a 512-channel data acquisition (DAQ) system. The physical mechanism underlying US contrast is illustrated in Fig. 1b. Incident US pulses excite phospholipid-shelled microbubbles (Sonovue), which undergo volumetric oscillations and emit secondary pressure waves that enable MC-US and ULM. The data-processing pipeline is summarized in Fig. 1c. Raw signals, split into subsets, were first bandpass-filtered with a Butterworth-type filter in the range of 0.2–8 MHz, followed by singular value decomposition (SVD) filtering to remove stationary tissue clutter and high-frequency noise. The cutoff eigenvector thresholds were empirically set to 50 for clutter suppression and 1000 for noise rejection. Reconstructed volumetric images were obtained via delay-and-sum (DAS) beamforming, and subsequent compounding via summation of the maxima of each subset and localization steps produced the MC-US and super-resolved ULM angiograms.

An analogous configuration was implemented for MC-OA and LOT imaging (Fig. 1d). In this case, a nanosecond-pulsed laser (<10 ns pulse duration) was coupled to an optical fiber bundle inserted through the central aperture of the array to deliver optical excitation to circulating, optically absorbing microcapsules [17]. Absorption of the optical pulse by the capsule shell induced thermoelastic expansion, generating acoustic waves that were detected by the same spherical array (Fig. 1e). OA data were processed using an identical computational pipeline (Fig. 1f). Signals were bandpass-filtered and SVD-cleaned using the same frequency and eigenvalue parameters as for the US data, then reconstructed via filtered back-projection (FBP). The reconstructed volumetric frames were subsequently normalized, compounded, and localized to yield motion-contrast optoacoustic (MC-OA) and super-resolved LOT images.

Although we refer to US and OA reconstruction as DAS and FBP, respectively, both methods are based on the same time-of-flight summation principle. In OA, the term FBP is commonly used as it is derived analogously to X-ray CT and includes a modality-specific temporal filtering step prior to DAS/backprojection. More advanced OA reconstructions (e.g., model-based or time-reversal) can reduce streak artefacts and increase apparent SNR, but may also change effective resolution and noise characteristics [24], [25]. To avoid modality-dependent advantages from different inversion strategies, we therefore used DAS-based reconstructions for both ULM and LOT so that differences primarily reflect the intrinsic contrast mechanisms.

## 3 Ultrasound emitter characterization and phantom imaging experiments

**Fig. 2:**
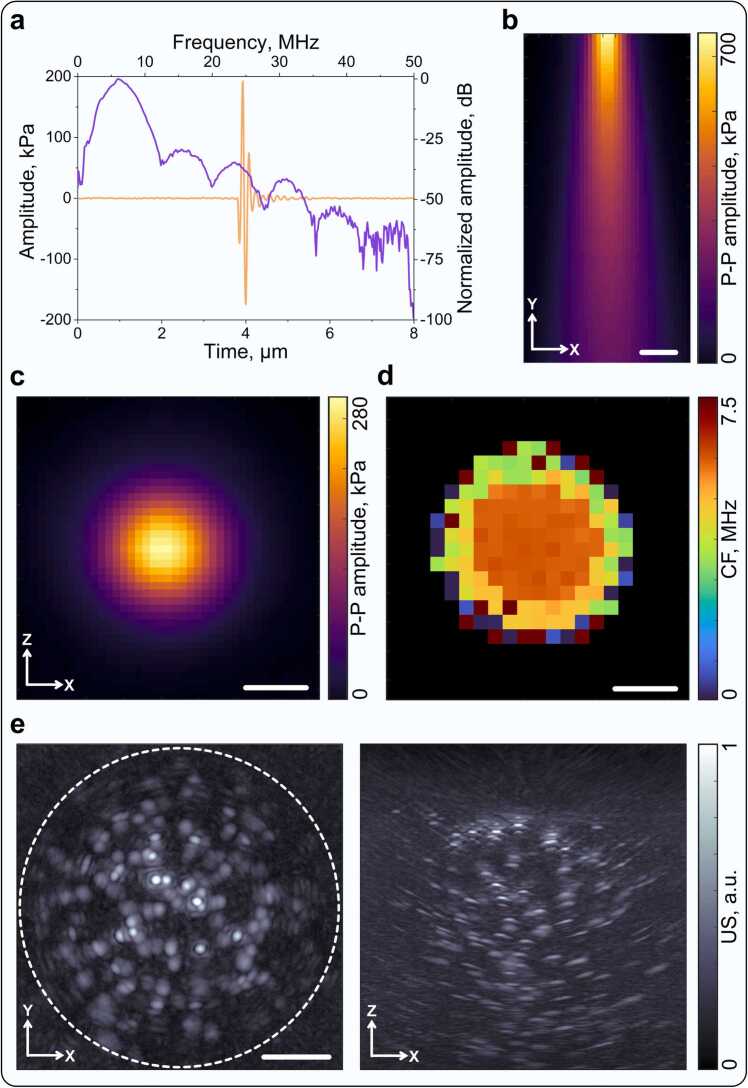
a) Characterized signal from the single-element transducer used for microbubble excitation in the experiments: time-domain signal and its Fourier transform. b) Axial peak-to-peak (P–P) pressure distribution measured at distances of 15–55 mm from the transducer. Scalebar: 5 mm. c) Transverse section of the peak-to-peak pressure amplitude at 40 mm distance from the transducer. d) Central frequency (CF) distribution within the ultrasound (US) beam at 40 mm distance from the emitting surface. Scalebars: 5 mm. e) Pulse-echo US image of 90 µm black polyethylene spheres embedded in agar with an optical scattering layer, showing the effective field of view (FOV) in two projections. Effective imaging circle: 10 mm diameter. Scalebars: 2 mm.

The spherical array transducer and microcapsules used in the experiments have been previously used for MC-OA/LOT [17]. However, the acoustic performance of the single-element US emitter used for microbubble excitation needs to be characterized to ensure controlled and reproducible delivery of acoustic energy within the imaging region (Fig. 2a-d). The emitted waveform exhibited a clean, single-cycle pulse in the time domain, with a well-defined spectral peak centered at approximately 7 MHz (Fig. 2a). This approximately matches the detection bandwidth of the spherical array transducer used in the experiments (Fig. S1a). Axial peak-to-peak (P–P) pressure mapping performed over distances of 15–55 mm from the emitter surface revealed the expected diverging propagation pattern, with pressure amplitude gradually decreasing as the beam broadened with distance (Fig. 2b). At a distance of 40 mm, corresponding to the curvature radius of the spherical ultrasound transducer and the position of the imaging target in subsequent experiments, the transverse pressure distribution exhibited a symmetric circular field with a maximum pressure amplitude of approximately 280 kPa (Fig. 2c). The corresponding central frequency (CF) map showed a homogeneous 7 MHz region in the beam core, while lower-frequency components became dominant toward the periphery, consistent with the broader angular propagation of lower-frequency US waves (Fig. 2d).

To further assess the imaging field of view (FOV) and overall system performance under controlled conditions, a tissue-mimicking phantom was prepared using 90 µm black polyethylene spheres embedded in a 1% agar matrix with mild optical scattering. The phantom was imaged using US excitation. The US images revealed a circular FOV of approximately 10 mm in diameter, clearly delineating the distribution of the embedded spheres in two orthogonal projections (Fig. 2e). The same phantom was also imaged in OA mode, showing a comparable FOV but with reduced imaging depth due to optical diffusion (Fig. S1b,c). Specifically, the OA mode mainly characterizes the reception-driven FOV of the spherical ultrasound array used in this study. The empirical PSF of the system was determined by imaging a single 50 µm black polyethylene sphere embedded in a 1% clear agar phantom (Fig. S1d,e). The measured PSF exhibits an approximately spherical shape in OA mode, whereas in US mode it shows a disk-like main lobe with symmetric axial side lobes above and below.

## 4 In vivo volumetric imaging of the mouse brain

**Fig. 3:**
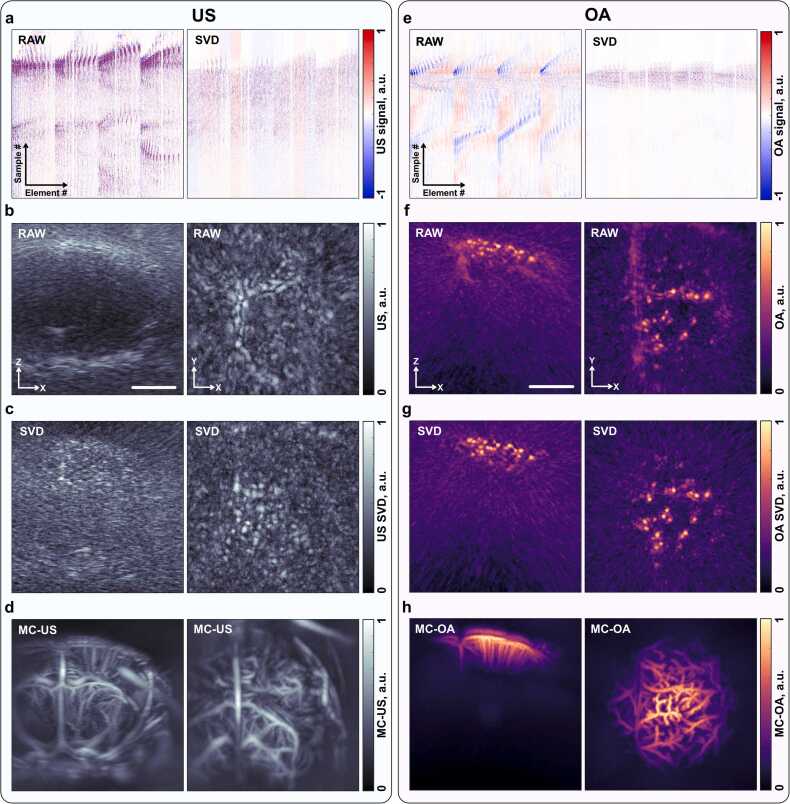
a) Typical sinogram of the recorded ultrasound (US) signals acquired with a spherical US transducer array using a through-aperture single-element transducer as the excitation source, shown before (RAW) and after (SVD) singular value decomposition filtering, with microbubbles circulating in the bloodstream. Vertical axis – recorded sample index; horizontal axis – spherical array transducer element index. b) Maximum intensity projections (MIPs) of the delay-and-sum (DAS) reconstructed 3D US image of the right hemisphere of the mouse cranium without SVD pre-filtering. Skull sutures are visible. Scalebar: 2 mm. c) MIPs of the DAS-reconstructed, SVD-filtered 3D US images showing microbubble signals. d) MIPs of MC-US images, accumulated over 5.5 min of acquisition at a 100 Hz frame rate. e) Typical sinogram of the recorded optoacoustic (OA) signal with the spherical ultrasound transducer using through-aperture pulsed laser excitation, shown before (RAW) and after (SVD) filtering, with microcapsules circulating in the bloodstream of the same mouse. f) MIPs of the filtered back-projection (FBP) reconstructed 3D OA image of the right hemisphere of the mouse cranium without SVD pre-filtering. Main vessels and microcapsules are visible. Scalebar: 2 mm. g) MIPs of the FBP-reconstructed, SVD-filtered 3D OA images showing isolated microcapsule signals. h) MIPs of MC-OA images, accumulated over 5.5 min of acquisition at a 100 Hz frame rate.

Volumetric *in vivo* imaging experiments were conducted on a young nude mouse (18 weeks old) to evaluate the performance of MC-US and MC-OA modalities using the unified acquisition setup described previously (Fig. 3). In the US experiments, a bolus of microbubbles was intravenously injected and acoustically excited using the US emitter inserted through the central aperture of the spherical array. The recorded raw US signals (sinogram) contained both dynamic microbubble signals and strong background clutter originating from stationary tissue and skull reflections (Fig. 3a, RAW). Application of SVD filtering effectively suppressed the static background, isolating signals arising from circulating microbubbles (Fig. 3a, SVD). Three-dimensional delay-and-sum (DAS) reconstruction with a voxel size of 40 µm produced volumetric images that clearly delineated skull sutures and surface reflections (Fig. 3b), whereas SVD-filtered reconstructions revealed distinct, spatially confined point sources corresponding to individual microbubbles within cerebral vessels, although these appeared partially blended into speckle-like patterns (Fig. 3c). Temporal accumulation of the SVD-filtered frames yielded MC-US maps, which emphasized dynamic vascular components and suppressed static tissues, resulting in high-contrast volumetric angiograms of the mouse brain (Fig. 3d).

A similar experiment was performed on the same mouse under identical conditions using the OA configuration, in which pulsed laser excitation was delivered coaxially through the array aperture. The corresponding OA sinogram (Fig. 3e, RAW) contained both static tissue background and transient signals from circulating microcapsules, which much like microbubbles in US were isolated following SVD filtering (Fig. 3e, SVD). Reconstruction via filtered back-projection (FBP) generated volumetric OA images of the right hemisphere of the mouse brain, where the main vascular branches and individual microcapsules were visible even without filtering (Fig. 3f). SVD-filtered reconstructions (Fig. 3g) further enhanced the contrast of isolated microcapsule signals, enabling clear delineation of the microvascular networks. Temporal accumulation of these reconstructed frames produced MC-OA images, highlighting the dynamic distribution of absorbing particles within the cerebral vasculature over 5.5 min of acquisition at a 100 Hz frame rate (Fig. 3h). The lateral width of microbubbles in US and microcapsules in OA was comparable between modalities and closely matched the dimensions obtained from microparticle measurements in phantom experiments. This observation indicates that the effective signal bandwidth is not substantially reduced under transcranial conditions of the young mouse. When MC-US and MC-OA results were compared side by side, a clear distinction in modality performance was observed. MC-US provided higher penetration depth, enabling full-brain visualization down to the circle of Willis, whereas MC-OA exhibited higher contrast and improved delineation of the superficial cortical vasculature.

## 5 Comparative performance in young and aged mouse brain imaging

**Fig. 4:**
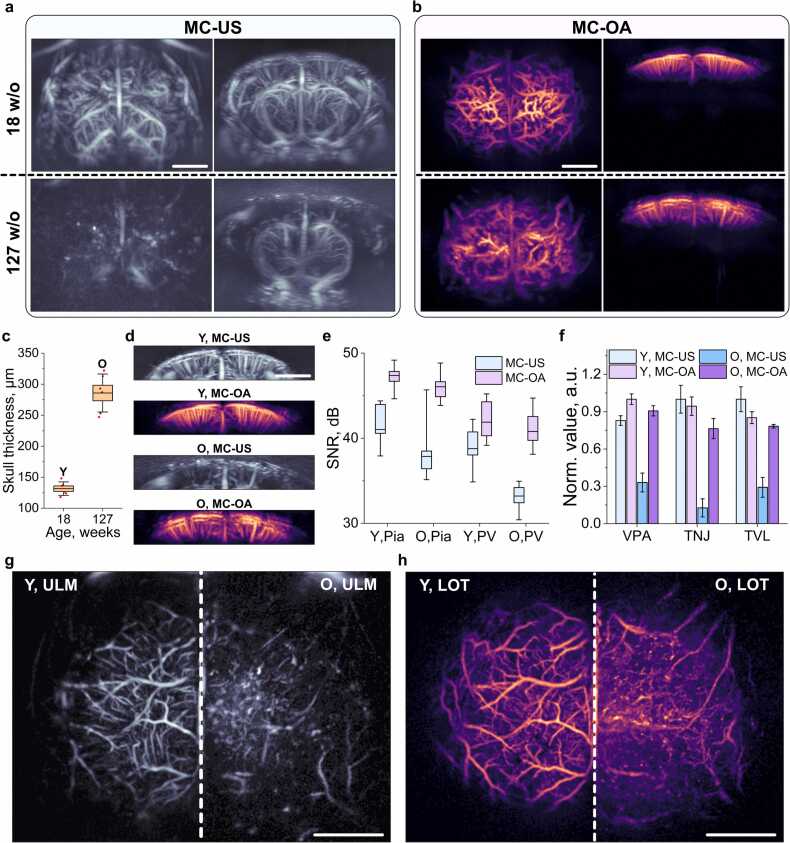
a) Combined motion-contrast ultrasound (MC-US) images of both hemispheres of a young mouse (18 w/o, upper row) and an old mouse (127 w/o, lower row) in two projections. b) Combined motion-contrast optoacoustic (MC-OA) images of both hemispheres of the same mice in two projections. Scalebars: 2 mm. c) Measured skull thickness in young (average age 18 weeks, 3 mice) and old (average age 127 weeks, 3 mice) specimens. Boxplot depicts 6 samples per group (3 mice, 2 hemispheres each); box borders indicate standard error, the central line indicates the mean, and whiskers represent standard deviation. Notation: 18 w/o – “Y”, 127 w/o – “O”. d) Isolated cortical images of young and old mice using MC-US and MC-OA modalities. Scalebar: 2 mm. e) Signal-to-noise ratio (SNR, in dB) in two cortical regions (Pia – pial vasculature; PV – penetrating vessels) in young and old mice (6 points in 3 hemispheres per group, 18 points per group in total), using MC-US and MC-OA modalities. The median is shown by the central line, box borders indicate quartiles, and whiskers represent minimum and maximum values. f) Vessel quantification of the cortex performed with AngioTool: vessel percentage area (VPA), total number of junctions (TNJ), and total vessel length (TVL). Bars indicate mean values; error bars represent standard deviation (SD). g) Side-by-side comparison of ULM cortical vascular maps of young (Y) and old (O) mice, reconstructed from accumulated localized microbubble positions. h) Side-by-side comparison of LOT cortical vascular maps of the same animals, reconstructed from accumulated localized microcapsule positions. Scalebars: 2 mm.

The imaging performance of MC-US/ULM and MC-OA/LOT modalities was systematically evaluated in young (18 weeks old) and aged (127 weeks old) mice to assess how skull properties and tissue composition influence image quality and vascular detectability (Fig. 4). Both modalities successfully captured the 3D cerebrovascular architecture of the mouse brain. However, their sensitivity and contrast profiles differed across age groups. In young mice, MC-US provided wide volumetric coverage with strong signal uniformity across both hemispheres, while MC-OA delivered higher contrast and finer vessel delineation in the superficial cortical layers (Fig. 4a,b). In aged mice, both modalities exhibited reduced vascular contrast, but the degradation was more pronounced in MC-US due to increased acoustic attenuation and wavefront distortion from the thickened skull. *Post-mortem* measurements confirmed a significant age-related increase in cranial thickness, supporting this observation with the young 18 weeks old calvaria with thickness of 130 μm, while the aged calvaria was 290 μm thick (Fig. 4c). Cropped images of the cortical region revealed that MC-OA retained higher signal stability and vessel visibility in aged mice (Fig. 4d). Quantitative comparison of the SNR in the pial (Pia, ∼0.5–1 mm from the skin surface) and penetrating-vessel (PV, ∼1–3.5 mm from the skin surface) regions showed that MC-OA achieved better SNR values in both age groups, indicating enhanced robustness to skull-induced attenuation (Fig. 4e). To further contextualize the depth limitations of the two modalities, simplified numerical simulations of optical and acoustic propagation through layered tissues were performed (Supplementary Methods). The simulations considered a four-layer model consisting of a water layer, skin, skull, and brain tissue. Monte Carlo photon transport simulations showed a progressive decay of optical fluence with depth due to scattering and absorption in tissue (Fig. S3). Increasing skull thickness had only a minor influence on the optical fluence distribution within the brain. In contrast, acoustic simulations demonstrated a more pronounced attenuation associated with the skull layer (Fig. S4), which becomes more pronounced for the round-trip propagation of US waves in ULM. To further compare the performance of both modalities, SNR was measured at six anatomically matched locations for each vessel type within every hemisphere (Fig. S2). To ensure statistical independence and reduce the dimensionality of the dataset, these measurements were averaged to obtain a single representative SNR value per hemisphere, modality, and vessel type. A 3-way ANOVA (analysis of variance) with age (Y vs. O), imaging modality (MC-OA vs. MC-US), and vessel type (Pia vs. PV) as factors revealed that imaging modality accounted for the largest fraction of the total variance (49.9%, p < 0.0001). Vessel type also contributed substantially (24.2%, p < 0.0001), while age had a smaller but still significant effect (13.7%, p = 0.0023). The results are presented in Table S1.

**Fig. 5:**
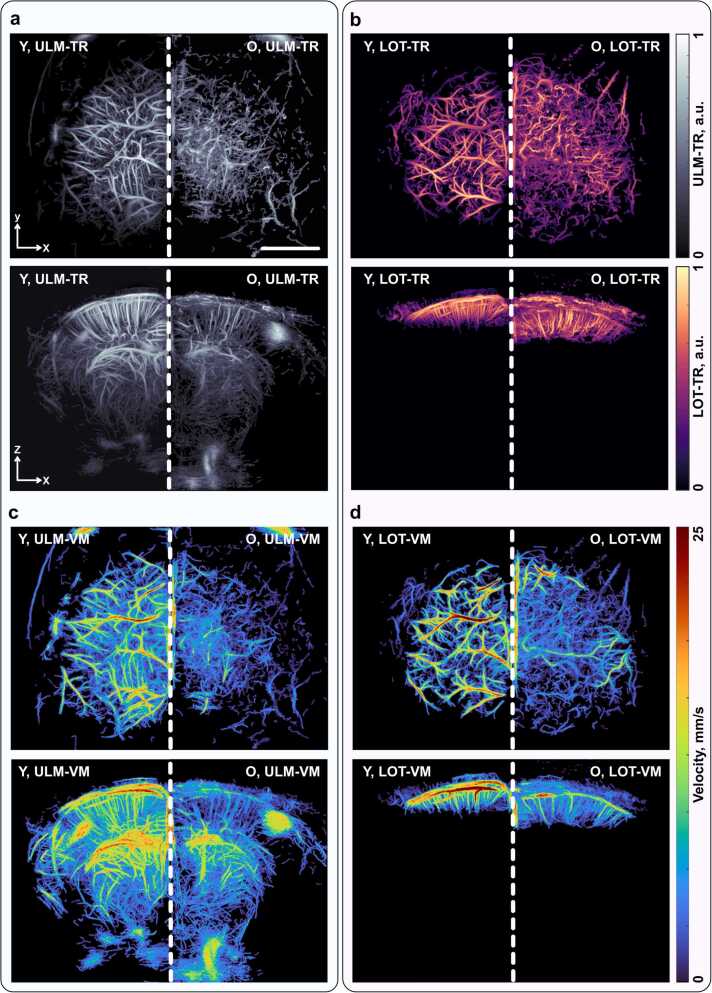
a) Side-by-side comparison of ULM images of young (Y) and old (O) mice shown in two projections, reconstructed from tracked microbubble trajectories. b) Side-by-side comparison of LOT images of the same young (Y) and old (O) mice in two projections, reconstructed from tracked microcapsule trajectories. c) Corresponding velocity maps obtained in ULM mode and d) LOT mode. Scalebar: 2 mm.

Vascular quantification further highlighted modality-specific differences between MC-US and MC-OA (Fig. 4f). In young animals, both modalities produced comparable vessel metrics, including vessel percentage area (VPA), total number of junctions (TNJ), and total vessel length (TVL). In older mice, however, MC-OA maintained higher relative values across all parameters, reflecting its improved robustness under conditions of increased acoustic attenuation. Statistical analysis was performed using a 1-way ANOVA followed by Tukey’s multiple comparisons test. For VPA, significant differences were observed between MC-US and MC-OA in both young (p = 0.0158) and old (p < 0.0001) animals. For TNJ, no significant difference was found between modalities in young mice (p = 0.8591), whereas a highly significant difference was observed in the old group (p < 0.0001). A similar trend was observed for TVL, with no significant difference in young mice (p = 0.1096) but a significant difference in older animals (p = 0.0001). Detailed results of all pairwise comparisons are provided in Table S2.

At the super-resolved level, ULM and LOT reconstructions provided detailed confirmation of these findings (Fig. 4g, h). ULM visualizations showed deeper but noisier coverage, while LOT maps displayed sharper definition of surface and mid-cortical vasculature (Fig. 4g,h and Fig. S5a). The effective spatial resolution of the reconstructions was quantified using Fourier Shell Correlation (FSC) analysis (Fig. S5b), yielding comparable sub-diffraction resolution of 32 µm for both modalities within the overlapping FOV. Together, these results demonstrate that MC-US/ULM excels in volumetric penetration, whereas MC-OA/LOT offers better sensitivity and vascular resolution in the optically and acoustically accessible cortical layers. The complementary performance of the two modalities across age groups underscores their combined potential for comprehensive cerebrovascular imaging.

Further processing of the datasets involved tracking the localized contrast agents using identical parameters for both modalities (see Materials and methods for more details). Instead of reconstructing vascular maps solely from accumulated localization points, the images were generated from filtered trajectories of the tracked particles. This trajectory-based reconstruction enables the formation of continuous vessel paths, improves the robustness of the vascular maps by suppressing isolated or spurious localization events that do not form consistent tracks, and facilitates subsequent hemodynamic analysis. In a subsequent step, velocity maps were derived from the tracked trajectories by estimating the displacement of the contrast agents along their paths. The resulting maps revealed comparable spatial patterns of blood flow in cortical regions where the FOV of the two modalities overlap. In both ULM and LOT reconstructions, the cortical vasculature exhibited similar flow organization, characterized by higher velocities in larger vessels and lower velocities within the microvascular network. A consistent reduction in flow velocities was observed in the old mice compared to the young animals in both modalities. This is consistent with previously reported age-related alterations in cerebral hemodynamics, where aging is associated with reduced cerebral blood flow and changes in microvascular perfusion [26]. Overall, the strong correspondence between the velocity patterns obtained with ULM and LOT further supports the consistency and complementarity of the two methods within their overlapping imaging regions.

## 6 Discussion and conclusion

OA and US imaging are natural partners, synergistically linked by their shared reliance on detecting time-resolved signals corresponding to US pressure waves. This facilitates hardware integration and reconstruction with analogous algorithms such as delay-and-sum or filtered back-projection. While early implementations capitalized on the practicality and ease of manufacturing of linear arrays, the tomographic nature of OA imaging and its inherent lack of speckle artifacts demands sufficient angular coverage to mitigate so-called limited-view artifacts [27], [28]. This requirement has driven the adoption of concave arrays, such as cylindrically-focused rings or hemispherical caps, which provide the necessary detection geometry for high-fidelity, OA reconstructions in two and three dimensions. Although such configurations are less conventional for pulse-echo US, they remain fully compatible, allowing for a direct and technically equitable comparison of the two modalities [29], [30]. Alternatively, arrays combining different parts (segments) have been proposed to facilitate hybridization and achieve optimal performance in both modalities [31], [32].

In this work, a spherical array was employed to establish a unified platform for benchmarking MC-US and MC-OA as well as super-resolution ULM and LOT. As both modalities rely on detecting signals from sub-resolution contrast agents, efficient imaging requires an excitation field matched to the sensitivity profile of the detection array and a large solid angle for efficient capture of weak acoustic responses. Both conditions are naturally satisfied by the spherical geometry, enabling an effective FOV of several millimeters per array position, with the penetration depth primarily dictated by optical and acoustic attenuation. This was sufficient to resolve cortical vascular networks with both modalities and enabled a direct comparison. Spherical arrays have previously been used for hybrid ULM/Power Doppler - OA imaging based on a synthetic aperture approach, where multiple array elements sequentially emit US to form a uniform probing beam [7], [33]. In contrast, the present implementation uses the array in receive-only mode, while US excitation is provided by a single transducer inserted through the central aperture. Although this configuration limits beam shaping, it substantially simplifies the acquisition hardware and avoids complex multi-channel transmit electronics. At the same time, the central emitter could be modified in frequency, size, or geometry in future implementations, offering additional flexibility for both imaging and therapeutic applications.

Ultrasound excitation at 7 MHz was chosen to match the detection bandwidth of the spherical array and maximize reconstruction fidelity. Despite transcranial propagation, the thin mouse skull (∼130 µm in young animals) did not significantly reduce the effective signal bandwidth, as evidenced by the comparable widths of reconstructed point sources in vivo. Although lower excitation frequencies (1–4 MHz) could improve penetration, they would primarily reduce spatial resolution, while the entire brain depth could already be imaged with the current configuration. In contrast, the penetration depth of LOT is mainly limited by optical attenuation rather than acoustic propagation.

A critical aspect of this comparative study was the adherence to safe exposure levels for US and light excitation pulses, which also reveals a fundamental operational difference between the modalities. For MC-US/ULM, the excitation energy is constrained by the mechanical fragility of the microbubbles [34], [35]. To prevent their destruction, the negative acoustic pressure must be maintained well below the regulatory mechanical index (MI) limit [36], [37], namely below approximately 500 kPa. For a bipolar pulse of 7 MHz central frequency, this corresponds to a transient acoustic energy density on the order of a few µJ/cm². In contrast, the primary safety consideration for MC-OA/LOT is thermal tissue damage. The laser fluence used in this work was maintained below the ANSI Z136.1 established safety limit of 35 mJ/cm² at 800 nm wavelength. Crucially, the light-absorbing microparticles employed by MC-OA/LOT are significantly more robust than microbubbles and remain undamaged at these fluence levels. This key distinction, where MC-US/ULM is constrained by agent fragility and MC-OA/LOT by tissue safety, partially explains the higher SNR observed with MC-OA/LOT in superficial cortical regions. The ability to safely employ higher excitation energy relative to the damage threshold of the particles allows MC-OA/LOT to generate stronger signals from microparticles near the brain surface, where optical attenuation is low. An additional limitation arises from safety constraints on the total deposited optical energy, which restrict the pulse repetition rate of the laser used for LOT. Moreover, tunable OPO lasers capable of delivering pulse energies on the order of tens of millijoules at pulse repetition rates exceeding ∼100–200 Hz need to be developed. Nevertheless, potential implementation of burst-mode pulse trains of high frequency could mitigate this issue. This limitation does not apply to ULM, which has already been successfully demonstrated in rodents with native three-dimensional acquisition at frame rates up to 750 Hz [38], [39], [40].

Obtained results are consistent with the expected and measured SNR differences for microbubbles signals in MC-US/ULM and microparticle signals in MC-OA/LOT. Indeed, the higher SNR in MC-OA/LOT at shallow depths directly reflected a finer vascular resolution of cortical microvasculature. However, the strong attenuation of light in biological tissues fundamentally limits its penetration. Conversely, MC-US/ULM capitalizes on the lower attenuation of US, providing exceptional visualization of deeper vascular networks in subcortical and deep-brain regions where optical methods are impeded. The performance gap between MC-OA/LOT and MC-US/ULM became more pronounced in aged mice. With advancing age, the murine skull undergoes continuous remodeling and deposition, leading to progressive thickening. The measured thickness of ∼290 µm in our study is consistent with previous reports for old mice [41], [42]. MC-US/ULM imaging of the rodent brain has generally required skull thinning or removal procedures, which are invasive and limit the feasibility of longitudinal studies [43]. In contrast, MC-OA/LOT has demonstrated the ability to accurately visualize cortical vasculature in a fully non-invasive manner. This capability is particularly relevant for murine models of neurodegenerative diseases, such as Alzheimer’s disease, which only manifest human-like pathological features at advanced ages [44]. By enabling repeated, non-invasive imaging, MC-OA/LOT offers a powerful tool to investigate cortical microvascular alterations associated with neurodegeneration.

With ongoing advancements in contrast agent design and excitation schemes, MC-OA/LOT and MC-US/ULM should not be viewed as mutually exclusive. Rather, they hold strong potential for complementary use, whether applied simultaneously or sequentially, to leverage the unique strengths of each modality. A combined approach could deliver high-SNR, high-resolution vascular imaging throughout the entire brain volume, including both cortex and deep structures, in a fully non-invasive manner. Such integration could be achieved through the adoption of hybrid excitation strategies [45], which enable co-registered or interleaved acquisition schemes that maximize the imaging capabilities of both modalities.

In summary, this work establishes a rigorous framework for comparing MC-US/ULM and MC-OA/LOT imaging of the murine brain. The comparative study performed demonstrates that MC-US/ULM and MC-OA/LOT should not be regarded as competing methods but as complementary modalities whose integration holds transformative potential. By leveraging the non-invasiveness and high resolution of MC-OA/LOT in the cortical areas with the depth penetration of MC-US/ULM, hybridization of the two techniques could achieve high-fidelity vascular mapping throughout the whole brain superior to the stand-alone approaches. Such combination would therefore significantly advance preclinical neurovascular research, particularly in aged and disease models, and may ultimately guide the design of translational imaging platforms for human applications.

## 7 Materials and methods

### 7.1 Materials

Agar, Intralipid 20%, and Tween 20 were obtained from Sigma Aldrich. Black polyethylene spheres (90 µm diameter) were purchased from Cospheric LLC, and microbubble preparation kits were procured from Bracco.

### 7.2 Imaging setup characterization

The pressure emitted by the single-element transducer (US emitter) was measured using a 1 mm diameter PVDF needle hydrophone (Precision Acoustics Ltd), connected to a preamplifier and DC coupler. Measurements were conducted in a thermally stabilized water tank preheated to 25 °C and filled with deionized water. The center of the emitter was aligned with the hydrophone, which was mounted on a three-axis motorized stage (IAI Inc.). Excitation pulses were generated by an ultrasound pulser-receiver (5073PR, Olympus), controlled via an arbitrary waveform generator (Rigol DG1022A, Rigol) operating at a pulse repetition rate of 100 Hz. The pressure signal at a distance of 40 mm from the emitter surface was recorded using a 12-bit single-channel waveform digitizer (ATS9351, Alazar Technologies Inc.) sampling at 100 MS/s. The axial pressure distribution was characterized by acquiring data along a horizontal slice measuring 25 × 40 mm², covering distances from 15 to 55 mm from the emitter surface. To characterize the pressure distribution perpendicular to the ultrasound beam propagation axis, the hydrophone was scanned in a raster pattern over a 25 × 25 mm² area with uniform 1 mm steps along both the x and y axes. The frequency profile of the beam was obtained by extracting the central frequency from the Fourier-transformed time-domain signal at each measurement point within the 25 × 25 mm² field.

### 7.3 Phantom imaging experiments

A tissue-mimicking phantom was prepared by mixing equal volumes (1:1) of 90 µm black polyethylene spheres suspended in a 0.1% Tween 20 aqueous solution with a 2% heated agar solution and Intralipid solution. The resulting suspension was gently mixed to avoid air bubble formation and rapidly poured into a cylindrical vessel (inner diameter: 25 mm), exceeding the required FOV for both imaging modalities. The vessel was placed in a −80 °C freezer for 5 min to promote rapid agar gelation and prevent vertical gradients in sphere concentration.A phantom containing a single 50 µm black polyethylene sphere was prepared in a similar manner, except that optical scattering was not included.

The solidified phantom was then mounted in a thermally stabilized water tank, with its flat surface facing a custom-built 512-element sparse spherical ultrasound array (Imasonic SaS) operating at a central frequency of 7 MHz. The array featured an 8 mm central aperture for coaxial excitation, and each element was connected to an independent channel of a 512-channel data acquisition system (Falkenstein Mikrosysteme GmbH) operating at 24 MS/s, providing 496 samples per element at a volumetric frame rate of 100 Hz. For US imaging, a 7 MHz single-element transducer was inserted into the central aperture of the array and used as the excitation source, while the array functioned as the receiver.

### 7.4 In vivo imaging experiments

MC-US and ULM imaging were performed using a coaxial ultrasound excitation scheme. A custom-made 7 MHz transducer with a 3 mm piezo crystal (Guangzhou Doppler Electronic Technologies Inc) was inserted into the central aperture of the spherical array. To minimize electromagnetic and acoustic crosstalk between excitation and reception, the emitter was positioned 40 mm from the array’s geometric focus. Excitation pulses were generated by an ultrasound pulser-receiver (5073PR, Olympus) controlled by an arbitrary waveform generator (Rigol DG1022A, Rigol).

The complete imaging assembly, including the array, central emitter or fiber bundle, and a water tank for acoustic coupling, was positioned above the mouse head. Ultrasound gel was applied between the tank and scalp to ensure efficient coupling.

For MC-OA and LOT, the fiber bundle was inserted through the array’s central aperture. A nanosecond-pulsed laser (EVO III, Innolas GmbH) operating at 800 nm was coupled to the bundle (NA = 0.5, Lightguide GmbH), delivering pulses at a fluence of 20 mJ/cm² - within safety limits for small-animal imaging. A 100 μL suspension of absorbing microcapsules was intravenously injected via a tail vein catheter over one minute, followed by a 6-minute acquisition to capture microcapsule dynamics. After LOT imaging, animals were allowed to rest for 30 min to ensure partial clearance of microparticles and prevent interference with subsequent ULM measurements. The microcapsules, being acoustically matched to water (water–thin shell–water structure), were not expected to produce significant US scattering.

For MC-US and ULM, the fiber bundle was replaced with the coaxial emitter for acoustic excitation. A 100 μL suspension of freshly prepared SonoVue microbubbles was injected through the same catheter, and ultrasound signals were recorded for 6 min. The volumetric acquisition rate for both modalities was 100 Hz.

After both imaging sessions, animals were euthanized in accordance with approved ethical protocols. Imaging was consistently performed in the sequence LOT first, followed by ULM, to minimize interference between contrast agents.

Four mice were used for imaging experiments to allow qualitative comparison: two young nude male mice (18 weeks old) and two aged wild-type male mice (127 weeks old), yielding three datasets (3 hemisphere imaging sessions). Post-mortem calvarial thickness was measured in each hemisphere using a digital dial indicator (ID-S IP42, Mitutoyo) in a total of six mice (three young, three old).

All animal procedures complied with the Swiss Federal Act on Animal Protection and were approved by the Cantonal Veterinary Office Zurich. Animals were housed in individually ventilated cages (IVCs) under specific pathogen-free (SPF) conditions. The housing environment was maintained at 20–24 °C and 45–65% relative humidity, with a 12-hour light/dark cycle. Mice had *ad libitum* access to standard rodent chow (3437PXL15, Cargill) and water. During all imaging procedures, anesthesia was induced with isoflurane (5% v/v) and maintained at 1.5% v/v (Abbott, Cham, Switzerland) in a medical oxygen/air mixture (100/400 mL/min). Anesthesia depth was continuously monitored, and body temperature was maintained using a heated platform to ensure physiological stability.

### 7.5 Data processing

Both MC-US/ULM and MC-OA/LOT datasets were processed using a unified pipeline to ensure a consistent basis for comparison. The raw datasets, comprising 496 samples × 512 transducer elements × 33000 time points, were first bandpass-filtered with a Butterworth-like filter (0.2–8 MHz) to suppress low-frequency drift and system noise. The filtered data were then divided into subsets of 1000 frames for sequential processing. To remove stationary tissue background and clutter, singular value decomposition (SVD) filtering was applied to each subset in the signal domain, with the first 50 eigenvectors removed. These parameters were empirically optimized to balance signal preservation and clutter rejection. Each filtered subset was subsequently reconstructed using GPU-accelerated algorithms: filtered backprojection for LOT and delay-and-sum beamforming for ULM, both rendered at a spatial resolution of 40 µm per voxel, yielding volumetric images of 200 × 200 × 200 voxels. From this point, the processing branched into two parallel analysis streams.

### 7.6 Motion-Contrast Imaging

Reconstructed images, split into multiple sets, were first used to compute the maximum of each set, and then summed over time to generate motion-contrast maps. These maps highlight dynamic components - such as flowing microbubbles in ULM or absorbing particles in LOT - while suppressing static background structures. Functionally, these representations are analogous to Power Doppler images, though terminology may vary depending on the reader’s background or interpretational framework.

### 7.7 Particle Localization

In each frame, moving microparticles were localized by correlating the signal with an empirically measured three-dimensional point spread function (PSF) of 7 × 7 × 7 voxels. Only detections with a correlation coefficient greater than 0.995 were retained to ensure high localization confidence. The centroid of each identified particle was then computed, and its spatial coordinates were recorded for every frame. Then, the localization images were constructed by plotting a point cloud of the localized central positions on a 3x-upsampled 3D grid, resulting in 600 × 600 × 600 voxels images. The resolution of both ULM and LOT was assessed using the Fourier Shell Correlation (FSC) method. Briefly, the SVD-filtered and localized signals were randomly divided into two independent datasets. Each dataset was then reconstructed separately to form independent ULM or LOT images. The FSC algorithm was subsequently applied to these image pairs, producing FSC curves as a function of the spatial frequency shell index, which was then converted to µm⁻¹ [46], [47].

Localized contrast agents were tracked to reconstruct particle trajectories and estimate blood flow velocities. Tracking was performed using the *simpletracker* algorithm (Tinevez et al., MATLAB implementation), which links localized detections between consecutive frames. The maximum linking distance was set to 6 pixels, and gap closing of up to two frames, extending the detectable velocity to ∼24 mm/s. Localization coordinates were initially obtained on a 40 µm reconstruction grid and subsequently upsampled (3 ×) prior to trajectory reconstruction to improve the spatial representation of particle paths. To suppress spurious detections and stationary noise, only trajectories satisfying predefined quality criteria were retained. Specifically, tracks containing fewer than three detections or with a total trajectory length shorter than 60 pixels were discarded. Blood flow velocities were estimated from the displacement of the tracked particles along their trajectories. To obtain smoother representations of particle motion, individual trajectories were interpolated and smoothed using spline fitting, with 80 interpolated points inserted between consecutive detections. Velocity values at each point were then computed from the displacement along the spline-fitted trajectories divided by the time interval determined by the pulse repetition rate. For consistency, the same tracking and velocity estimation parameters were applied to both the ULM and LOT datasets.

### 7.8 Image analysis

The SNR of the MC-US and MC-OA images was quantified using manually selected regions of interest (ROIs) in the pial and penetrating vessel areas. Selections were made in three hemispheres, with six points per region, resulting in 18 measurements per group for both young and old mice. The SNR (in dB) was calculated according to the equation:

$${\mathrm{SNR}}_{\mathrm{dB}}=20\times \log_{10}\;\left(\;\frac{\mu_{\mathrm{Signal}}}{\sigma_{\mathrm{Noise}}\;}\right),$$

where $\mu_{\mathrm{Signal}}$ is the mean signal intensity within a 2-pixel radius around the selected point, and $\sigma_{\mathrm{Noise}}$ represents the standard deviation of the noise calculated within a 10 × 10 × 10 voxel region placed in a corner of the reconstructed volume outside the mouse brain. The resulting SNR values were summarized using box plots, where the central line indicates the median, box borders represent the interquartile range, and whiskers denote the minimum and maximum values.

For quantitative vessel analysis, AngioTool v0.6 was used to extract vascular morphology parameters, including vessel percentage area (VPA), total number of junctions (TNJ), and total vessel length (TVL). These metrics were computed for three datasets per age group (young and old). The results were visualized as bar plots, with bar height indicating the mean value and error bars representing the standard deviation (SD).

SNR was quantified at six anatomically matched regions of interest per vessel type within each hemisphere. To avoid pseudoreplication, measurements from the six regions were averaged, yielding a single representative value per hemisphere for each modality and vessel type. Statistical evaluation of SNR was performed using a 3-way ANOVA with age (young vs. old), imaging modality (MC-US vs. MC-OA), and vessel type (pial vs. penetrating) as factors. Vascular network metrics, including vessel percentage area (VPA), total number of junctions (TNJ), and total vessel length (TVL), were quantified from the reconstructed angiograms. Differences between experimental groups (young MC-US, young MC-OA, old MC-US, and old MC-OA) were assessed using a 1-way ANOVA followed by Tukey’s multiple comparisons test. Statistical analyses were performed using Prism Graphpad 10.0.3, and p < 0.05 was considered statistically significant.

## Declaration of generative AI and AI-assisted technologies in the manuscript preparation process

During the preparation of this work the authors used ChatGPT 40 in order to improve the coherence of the text. After using this tool/service, the authors reviewed and edited the content as needed and take full responsibility for the content of the published article.

## CRediT authorship contribution statement

**Elshad Feyzili:** Investigation, Methodology, Writing – review & editing. **Cristian Ciobanu:** Investigation, Methodology, Writing – review & editing. **Xosé Luís Deán-Ben:** Writing – original draft, Supervision, Project administration, Methodology, Formal analysis, Conceptualization. **Daniel Razansky:** Writing – original draft, Supervision, Resources, Funding acquisition. **Yi Chen:** Writing – original draft, Visualization, Methodology, Investigation, Data curation. **Daniil Nozdriukhin:** Writing – original draft, Visualization, Methodology, Investigation, Formal analysis, Conceptualization.

## Declaration of Competing Interest

The authors declare that they have no known competing financial interests or personal relationships that could have appeared to influence the work reported in this paper.

## Data Availability

Data will be made available on request.
