# Supplementary material for: Three-dimensional microangiography of the mouse brain using super-resolution ultrasound and optoacoustic imaging with a spherical array transducer
Source: Photoacoustics. 2026 Mar 28;49:100823. doi: 10.1016/j.pacs.2026.100823 (PMC13087747; doi:10.1016/j.pacs.2026.100823)
Supplement: Supplementary file 1 — Supplementary material [file mmc1.docx]

**Supplementary Information**

**Three-dimensional microangiography of the mouse brain using super-resolution ultrasound and optoacoustic imaging with a spherical array transducer**

Daniil Nozdriukhin ^1,2^, Yi Chen ^1,2^, Cristian Ciobanu ^1,2^, Elshad Feyzili ^1,2^, Daniel Razansky ^1,2^, Xosé Luís Deán-Ben ^1,2,*^

^1^Institute for Biomedical Engineering and Institute of Pharmacology and Toxicology, Faculty of Medicine, University of Zurich, Winterthurerstrasse 190, Zurich, 8057 Switzerland

^2^Institute for Biomedical Engineering, Department of Information Technology and Electrical Engineering, ETH Zurich, Wolfgang-Pauli-Strasse 27, Zurich, 8093 Switzerland

*Corresponding author. E-mail: [xl.deanben@pharma.uzh.ch](mailto:xl.deanben@pharma.uzh.ch)


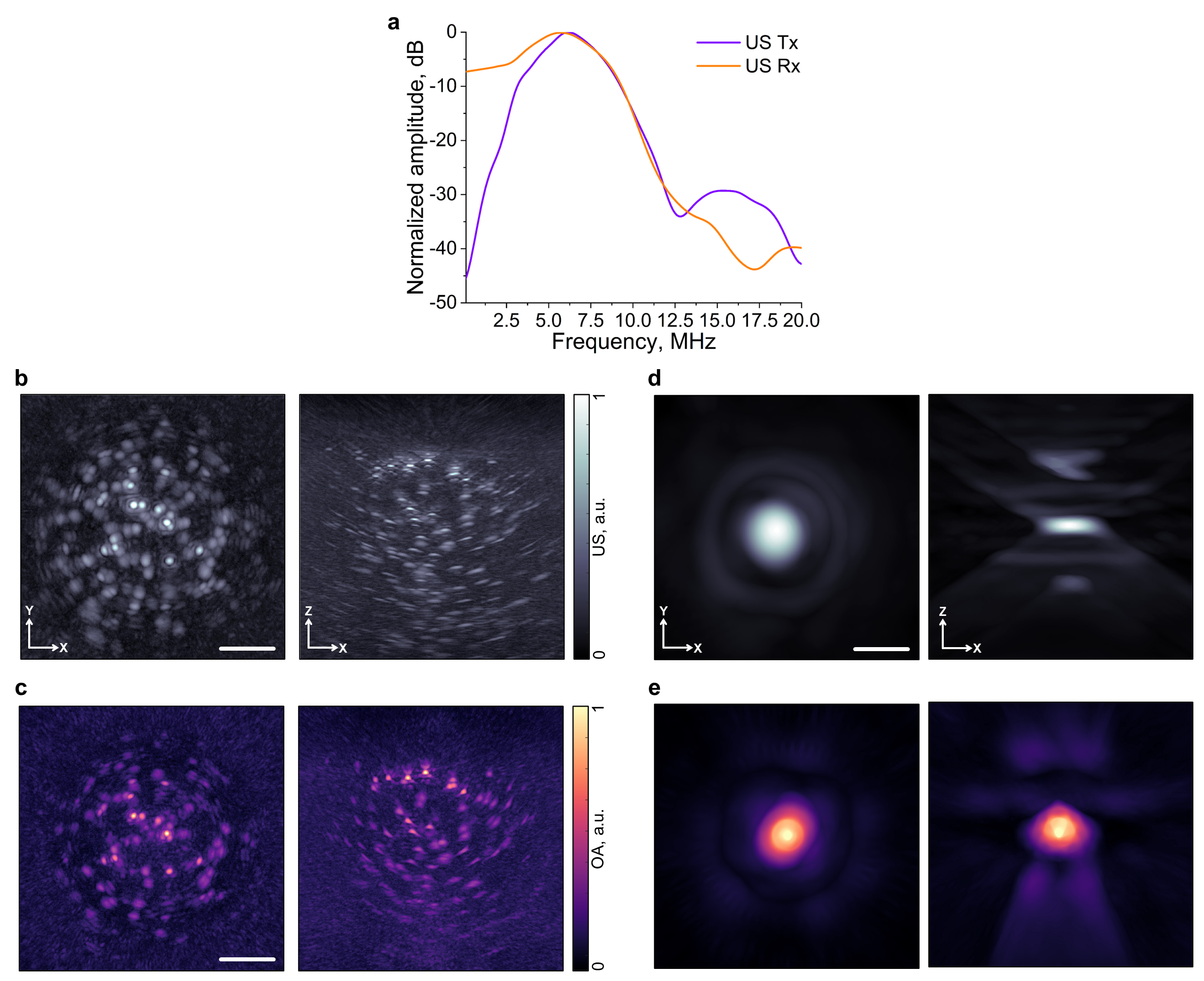


**Figure S1.** a) Comparison of transmission (Tx) bandwidth of the US emitter and reception (Rx) bandwidth of the spherical array transducer, used in study. b) US image of 90 µm black polyethylene spheres embedded in agar with an optical scattering layer from Fig. 2 of the manuscript and c) the same phantom, imaged in OA mode, demonstrating comparable FOV. Scalebars: 2 mm. d) Point-spread function (PSF) in US and e) OA modes, acquired by imaging a subwavelength 50 µm black polyethylene sphere, embedded in a clear agar. Scalebar is 200 µm.


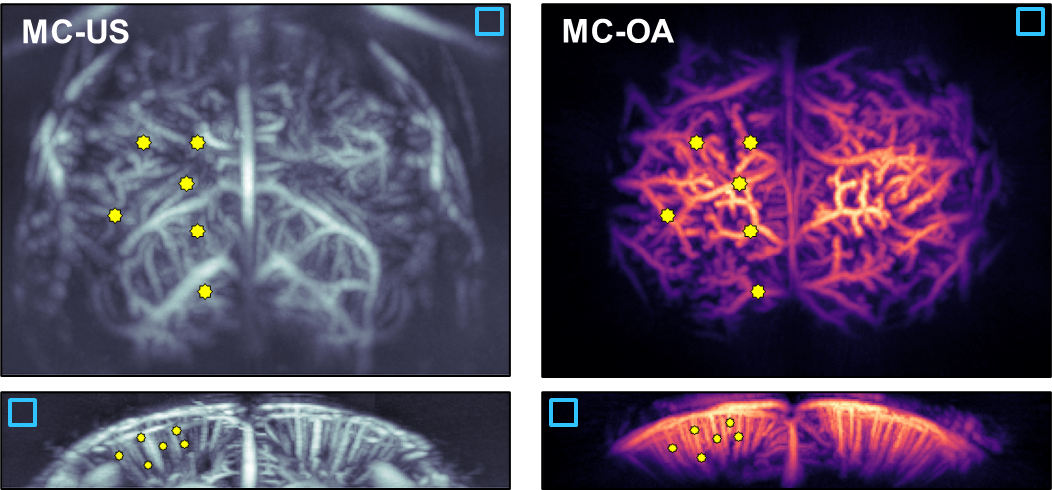


**Figure S2.** An example of points for SNR calculation: blue squares indicate the noise calculation area, 10 × 10 × 10 voxels, outside of the brain area. Yellow dots indicate the approximate distribution of point in pial vasculature and penetrating vessels, 2 × 2 × 2 voxels.

**Table S1:**

Statistical analysis of SNR data from Figure 4e using a 3-way ANOVA test.

| Source of Variation | % of total variation | P value | Result |
| --- | --- | --- | --- |
| Method (MC-OA vs MC-US) | 49.91 | <0.0001 | **** |
| Vessel type (pial vs penetrating vessels) | 24.16 | <0.0001 | **** |
| Age (young vs old) | 13.66 | 0.0023 | ** |

**Table S2:**

Statistical analysis (in-group) of vessel metrics data from Figure 4f using 1-way ANOVA with Tukey's multiple comparisons test. Y – young, O – old.

| Metric | Tukey's multiple comparisons test | Result | Adjusted P Value |
| --- | --- | --- | --- |
| VPA | Y, MC-US vs. Y, MC-OA | * | 0.0158 |
|  | Y, MC-US vs. O, MC-US | **** | <0.0001 |
|  | Y, MC-US vs. O, MC-OA | ns | 0.3231 |
|  | Y, MC-OA vs. O, MC-US | **** | <0.0001 |
|  | Y MC-OA vs O, MC-OA | ns | 0.2037 |
|  | O, MC-US vs O, MC-OA | **** | <0.0001 |
|  |  |  |  |
| TNJ | Y, MC-US vs. Y, MC-OA | ns | 0.8591 |
|  | Y, MC-US vs. O, MC-US | **** | <0.0001 |
|  | Y, MC-US vs. O, MC-OA | * | 0.041 |
|  | Y, MC-OA vs. O, MC-US | **** | <0.0001 |
|  | Y MC-OA vs O, MC-OA | ns | 0.1239 |
|  | O, MC-US vs O, MC-OA | **** | <0.0001 |
|  |  |  |  |
| TVL | Y, MC-US vs. Y, MC-OA | ns | 0.1096 |
|  | Y, MC-US vs. O, MC-US | **** | <0.0001 |
|  | Y, MC-US vs. O, MC-OA | * | 0.0195 |
|  | Y, MC-OA vs. O, MC-US | **** | <0.0001 |
|  | Y MC-OA vs O, MC-OA | ns | 0.6219 |
|  | O, MC-US vs O, MC-OA | *** | 0.0001 |

**Supplementary materials and methods**

To estimate signal attenuation under the experimental conditions of this study, simplified numerical simulations of optical and acoustic wave propagation through layered tissues were performed. The simulated geometry consisted of four layers representing the tissues encountered during in vivo imaging: water (2 mm), skin (500 µm), skull (130 µm or 290 µm), and brain tissue (10 mm). The model assumed planar interfaces between layers and homogeneous optical and acoustic properties within each region.

**Optical propagation simulations**

Optical photon propagation was simulated using the Monte Carlo eXtreme (MCX) GPU-accelerated Monte Carlo photon transport package. MCX solves the radiative transfer equation using stochastic photon propagation and is widely used for modeling light transport in scattering biological tissues.

The computational domain was discretized using a uniform voxel grid with isotropic resolution of 20 µm in all spatial directions. The total simulated domain size was 500 voxels the axial dimension and 500 voxels in the lateral dimension. The tissue layers were assigned to the voxel grid according to their physical thickness. Each tissue layer was assigned optical parameters including absorption coefficient (μ_a_), reduced scattering coefficient (μ_s_′), anisotropy factor (g), and refractive index (n). Optical parameters were selected from literature values reported for biological tissues in the near-infrared wavelength range (800 nm where available) commonly used in optoacoustic imaging. The illumination source was modeled as a Gaussian-like planar photon source positioned in the water layer and propagating toward the tissue surface. The source had a Gaussian beam profile with an 8 mm full width at half maximum (FWHM) and normal incidence relative to the tissue surface. A total of 10⁸ photons were launched in each simulation to ensure stable fluence estimation. The spatial distribution of optical fluence inside the tissue was recorded and used to evaluate attenuation with depth. The resulting fluence maps and centerline profiles are shown in Fig. S3.

**Acoustic propagation simulations**

Ultrasound propagation was simulated using the k-Wave MATLAB toolbox, which solves the acoustic wave equation using a pseudospectral time-domain method. The acoustic simulation grid consisted of 640 axial grid points and 500 lateral grid points, with isotropic spatial resolution of 20 µm. Each tissue layer was assigned acoustic parameters including speed of sound, density, and acoustic attenuation, using values reported in the literature for soft tissues and cortical bone. The ultrasound excitation was modeled as a Gaussian-distributed velocity source positioned in the water layer above the tissue surface. The beam profile was defined by a Gaussian envelope with a beam waist of 3 mm, corresponding to the experimental transducer configuration. The simulated frequency range was 5-9 MHz, with a central frequency of 7 MHz. The simulated acoustic field propagated through the layered medium, and the transmitted pressure field was recorded inside the brain layer to estimate attenuation caused by the skull layer. The resulting pressure transmission profiles for different skull thicknesses are shown in Fig. S4.


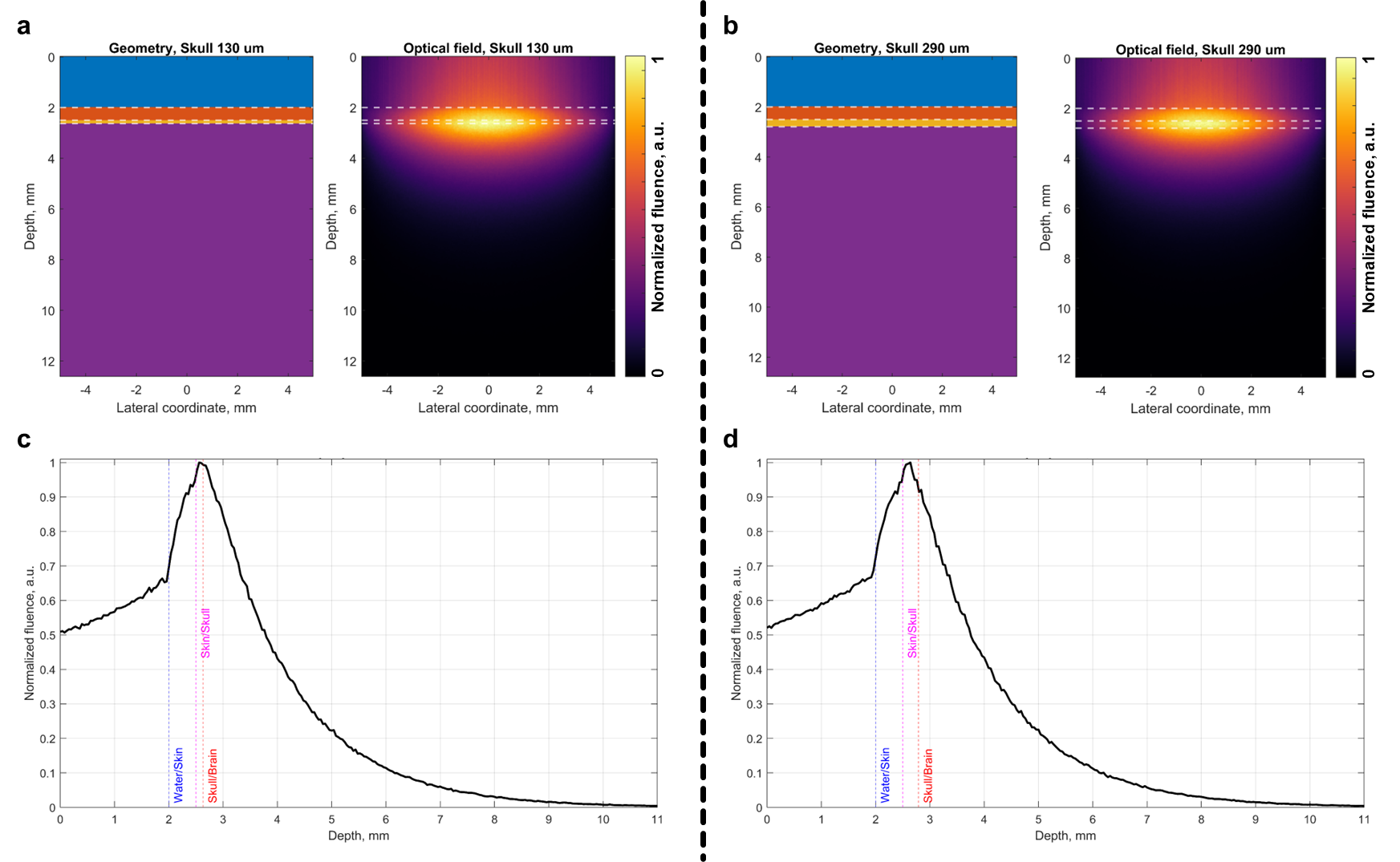


**Figure S3.** a) Left panel - layered geometry used for the Monte Carlo photon propagation simulations with 20 µm uniform grid. From top to bottom: water (2 mm, blue), skin (500 µm, orange), skull (130 µm, yellow), and brain (10 mm, purple). The colours are illustrative and not related to the optical properties of the media. Right panel - light fluence distribution of (10^8^) photons launched from a Gaussian-like source with an 8 mm FWHM in water. b) Left panel - layered geometry used for the Monte Carlo photon propagation simulations. From top to bottom: water (2 mm, blue), skin (500 µm, orange), skull (290 µm, yellow), and brain (10 mm, purple). The colours are illustrative and not related to the optical properties of the media. Right panel-light fluence distribution of (10^8^) photons launched from a Gaussian-like source with an 8 mm FWHM in water. c) Fluence profile along the centerline of the optical field (lateral coordinate = 0), showing the fluence decay in the brain for a 130 µm skull (young mouse). d) Fluence profile along the centerline of the optical field (lateral coordinate = 0), showing the fluence decay in the brain for a 290 µm skull (old mouse).


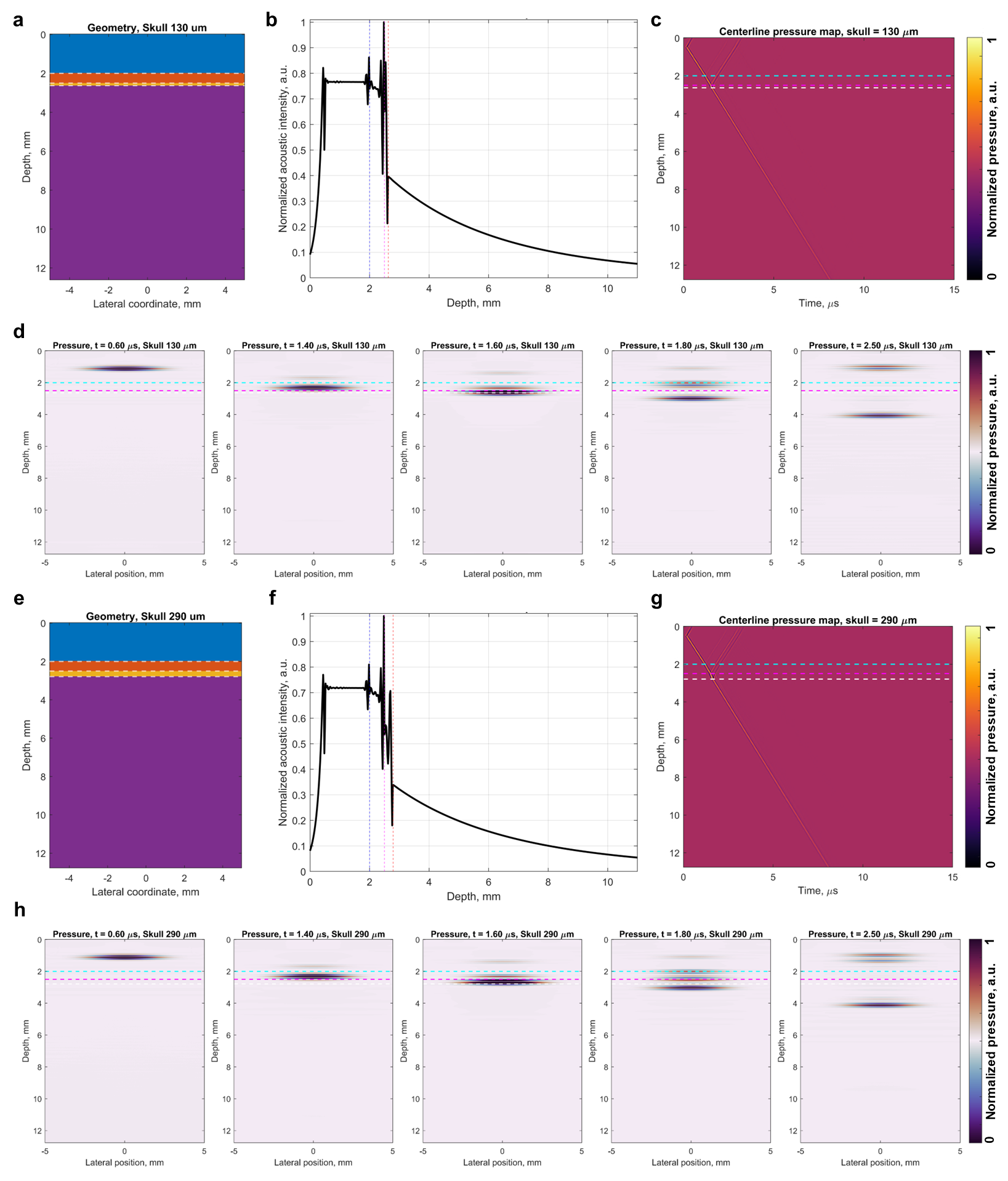


**Figure S4.** a) Layered geometry used for the k-Wave ultrasound propagation simulations with a 20 µm uniform grid. From top to bottom: water (2 mm, blue), skin (500 µm, orange), skull (130 µm, yellow), and brain (10 mm, purple). The colors are illustrative and do not correspond to the acoustic properties of the media. b) Acoustic intensity profile along the centerline of the geometry generated by a Gaussian-like source with a 3 mm FWHM aperture. Vertical lines indicate the interfaces between media (water-skin, skin-skull, and skull-brain; see Fig. S3). c) Spatiotemporal evolution of the centerline pressure in the model containing a 130 µm skull (young mouse). d) Snapshots of the pressure wave propagation through the layered model: before interaction with tissue, after reflection and transmission at the water-skin interface, after interaction with the skin-skull interface, after secondary reflections between the skin-skull and skull-brain interfaces, and during propagation within the brain tissue. e) Layered geometry used for the k-Wave ultrasound propagation simulations with a 20 µm uniform grid, with the skull thickness increased to 290 µm to represent an older mouse. From top to bottom: water (2 mm, blue), skin (500 µm, orange), skull (290 µm, yellow), and brain (10 mm, purple). Colors are illustrative and do not correspond to acoustic properties. f) Acoustic intensity profile along the centerline of the geometry generated by the Gaussian-like source with a 3 mm FWHM aperture. Vertical lines indicate the interfaces between media (water–skin, skin–skull, and skull-brain; see Fig. S3). g) Spatiotemporal evolution of the centerline pressure in the model with a 290 µm skull (old mouse). h) Snapshots of pressure wave propagation through the layered model illustrating the same stages as in (d): prior to tissue interaction, after reflection and transmission at the water-skin interface, after interaction with the skin-skull interface, after secondary reflections between the skin-skull and skull-brain interfaces, and during propagation in the brain tissue.


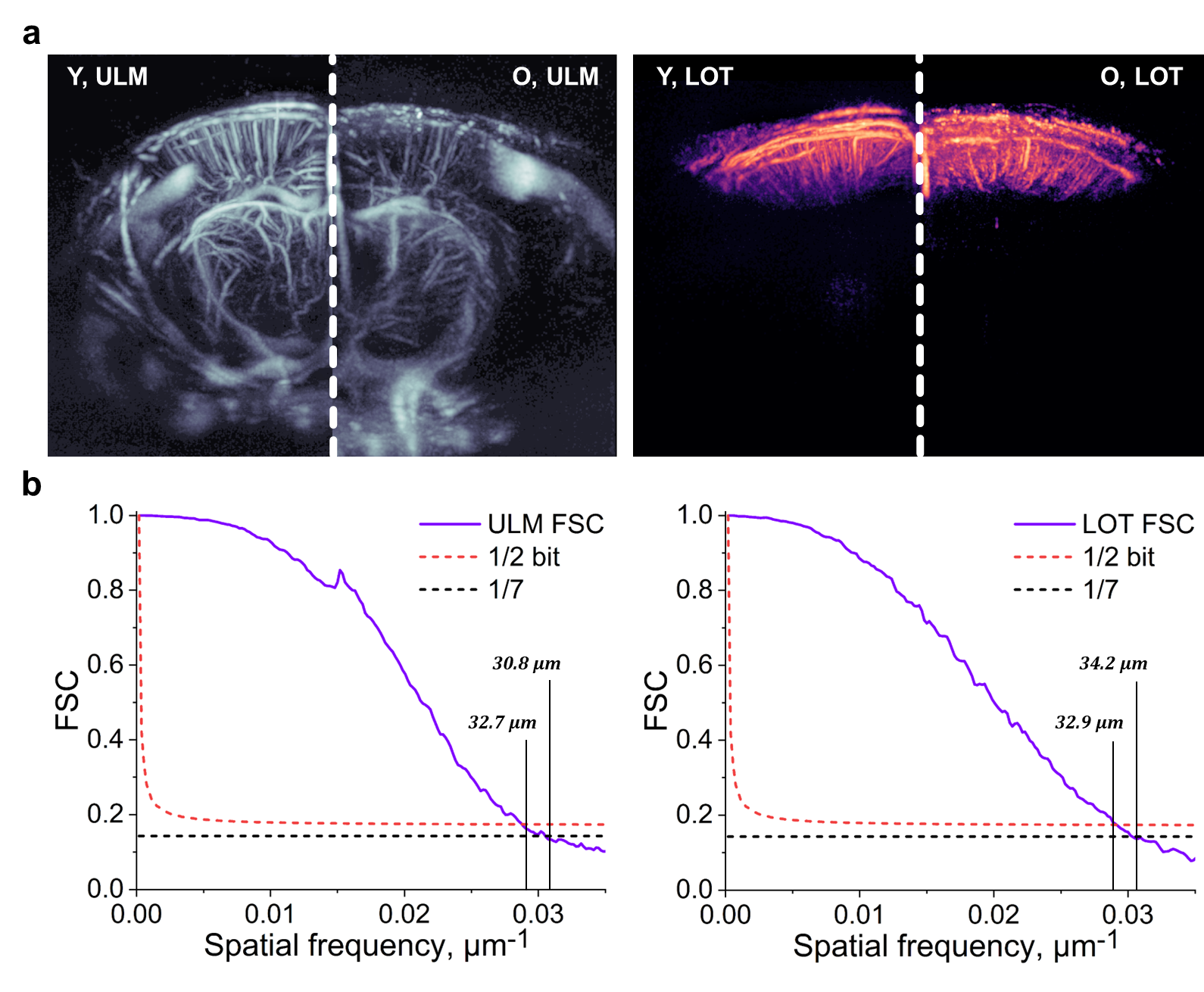


**Figure S5.** a) Coronal MIP views of ULM and LOT for young (Y) and old (O) mouse b) Results of FSC analysis of ULM and LOT datasets.
